# The prevalence of depression among adolescent with HIV/AIDS: a systematic review and meta-analysis

**DOI:** 10.1186/s12981-021-00351-1

**Published:** 2021-04-27

**Authors:** Getinet Ayano, Sileshi Demelash, Mebratu Abraha, Light Tsegay

**Affiliations:** 1 Research and Training Department, Amanuel Mental Specialized Hospital, Addis Ababa, Ethiopia; 2 School of Public Health, Curtin University, Perth, WA Australia; 3 Department of Psychiatry, Paulo’s Millennium Medical College, Addis Ababa, Ethiopia; 4 Ethiopian Public Health Institute, Addis Ababa, Ethiopia; 5 Departmemt of Psychiatry, Aksum University, Aksum, Ethiopia

**Keywords:** Depression, Adolescent, HIV, AIDS, Systematic review, Meta-analysis

## Abstract

**Background:**

Depression is particularly common among adolescents with HIV/AIDS and has been associated with disruption of the important developmental process, subsequently leading to a wide range of negative mental, physical and psychosocial consequences, as well as poor quality of life in those population groups. Nevertheless, to the best of our knowledge, there are no prior systematic reviews and meta-analytic studies that determined the prevalence of depression among adolescents with HIV/AIDS.

**Method:**

We systematically searched PubMed, Scopus and Web of Science for relevant literature until May 2020. A random-effect meta-analysis was used to pool prevalence rates from individual studies. Sensitivity and subgroup analyses were performed to identify the source of heterogeneities and to compare the prevalence estimates across the groups. The Joanna Briggs Institute’s quality assessment checklist was used to evaluate the quality of the included studies. Cochran’s Q and the I^2^ tests were used to assess heterogeneity between the studies.

**Results:**

A total of ten studies were included for the final analysis, with 2642 adolescents living with HIV/AIDS. Our final meta-analysis showed that more than a quarter of adolescents with HIV had depression [26.07% (95% CI 18.92–34.78)]. The prevalence was highest amongst female adolescents (32.15%) than males (25.07%) as well as amongst the older adolescents aged 15–19 years (37.09%) than younger adolescents aged 10–14 years (29.82%).

**Conclusion:**

Our study shows that a significant proportion of adolescents with HIV had depression, indicating the imperativeness of intervention strategies to alleviate the suffering and possibly reduce the probable negative ramifications.

**Supplementary Information:**

The online version contains supplementary material available at 10.1186/s12981-021-00351-1.

## Background

Studies have suggested that roughly 2.1 million (95% confidence interval of 1.4–2.7 million) adolescents aged 10–19 years are living with HIV/AIDS globally [1, 2]. Evidence shows that depression is highly prevalent among adolescents living with HIV/AIDS when compared with those adolescents without HIV/AIDS [3–6]. In general, the reported prevalence of depression among adolescents with HIV/AIDS varies across the studies from 11.40 to 45.83% [3–10]. There are several factors, which are responsible for this significant difference in the prevalence of depression including (i) the variations in the characteristics of the adolescents across the studies regarding the severity of HIV infections, the stages of the disease, as well as the presence of opportunistic infections; (ii) the differences in the tools used to measure depression between the studies with various psychometric properties including sensitivity and specificity; (iii) the variation in clinical characteristics of participants including the presence of other mental health problems as well as additional psychosocial stress or trauma among the participants.

Epidemiological studies conducted in the past several decades shows that the presence of depression among adolescents living with HIV/AIDS has been consistently associated with disruption of the important developmental process, subsequently leading to a wide range of negative mental, physical and psychosocial consequences, as well as poor physical health in those population groups [11, 12]. Complementing the above views, scientific studies revealed that the presence of depression among adolescents with HIV is linked with poor quality of life [12, 13], increased levels of stigma [13–15], poor social support [12], suicide [16], as well as a poor coping mechanism leading to a wide range of negative physical health problems and difficulties in managing social aspects of life [17]. Moreover, an emerging body of scientific evidence suggests that depression in early life has been linked with poor educational attainment, unemployment, as well as lower levels of perceived social sports (subjective judgments about the availability of help from friends and family during the times of need) [18–20]. Moreover, the presence of depression among adolescents living with HIV/AIDS is also linked with poor adherence to antiretroviral treatment(ART), which in turn associated with significant morbidity, mortality, increased resistance to ART drugs, severe disease, lower quality of life and short life expectancy due to untreated or inadequately treated problems [21–24]. Therefore, understanding the true magnitude of depression among adolescents with HIV is imperative for implementing possible early screening and suitable intervention strategies, subsequently reducing the suffering as well as the associated negative outcomes.

No systematic review and meta-analysis to date have estimated the consolidated prevalence of depression among adolescents living with HIV/AIDS. Evidence from such meta-analysis will provide robust information on the epidemiology of depression among adolescents with HIV/AIDS that would be necessary to plan early and suitable intervention strategies for those population groups. Therefore, the purpose of this review is to systematically analyze published studies on the prevalence of depression among adolescents with HIV/AIDS using both qualitative and quantitative methods.

## Research design and method

This systematic review and meta-analysis was designed, conducted and reported in accordance with the preferred reporting items for systematic reviews and meta-analyses (PRISMA) guidelines [25]. The systematic searching, assessing eligibility, evaluating the quality, extracting data and analysis of data has been performed based on predesigned protocol.

### Data source and selection process

We searched PubMed, Scopus and Web of Science to identify relevant studies published in the English language until May 2020. The following keywords and terms were used to assess PubMed: [(depression or depressive symptom or mental health) and (adolescent or child or youth)] and (Human immunodeficiency virus or HIV or acquired immunodeficiency syndrome or AIDS). Scopus and Web of Science were searched using terms and words suitable for the databases. Additional relevant studies were collected by searching through the reference lists of eligible studies (Additional file 1: Fig. S1).

### Eligibility criteria and study selection

Retrieved papers were included in this review if they satisfy the following criteria: (i) The study participants were adolescents with HIV/AIDS; (ii) reported the prevalence rates of depression or reported the data for calculating the prevalence and (iii) published in the English language. Reviews, commentaries, case reports and articles performed on animal subjects were excluded. Further, letters to the editor, conference papers, books, editorials and notes were also excluded from the study.

### Methods for data extraction and quality assessment

Two independent authors (MA and GA) extracted relevant data from the included studies. The data extracted per study included the following information: first author(s) name, sample size, year of publication, the country where the study was conducted, the tools used to assess depression, and the number of positive cases and the corresponding prevalence estimates. We also extracted the number of positive cases and the prevalence rates specifically for male and female participants as well as for the older and younger adolescents.

The Joanna Briggs institute quality assessment tool was employed to evaluate the quality of studies included in the final analysis [26]. The scoring of individual studies was conducted according to the frequency scales that are answered as yes, no, not clear and not applicable. To calculate the total quality score for each study we have utilized the total numbers of positive scores.

### Data synthesis and analysis

In this study, all statistical analyses were conducted by using the comprehensive meta-analysis software version 3 [27]. The prevalence rates from the individual studies were pooled by using a random-effect meta-analysis [28]. The I^2^ statistics have been used to assess the heterogeneity between studies [28]. The values of *I*^2^ statistics such as 75, 50 and 25%, represented high medium and low heterogeneity respectively [29]. The gender of the participants, age, the countries and the quality of the studies were used to evaluate the possible source of heterogeneity across the studies. Egger’s regression tests and funnel plots were used to measure the risk of publication bias. The P-value for statistical significance was set at 0.05 for all analyses.

## Results

### Identification of relevant studies

**Fig. 1:**
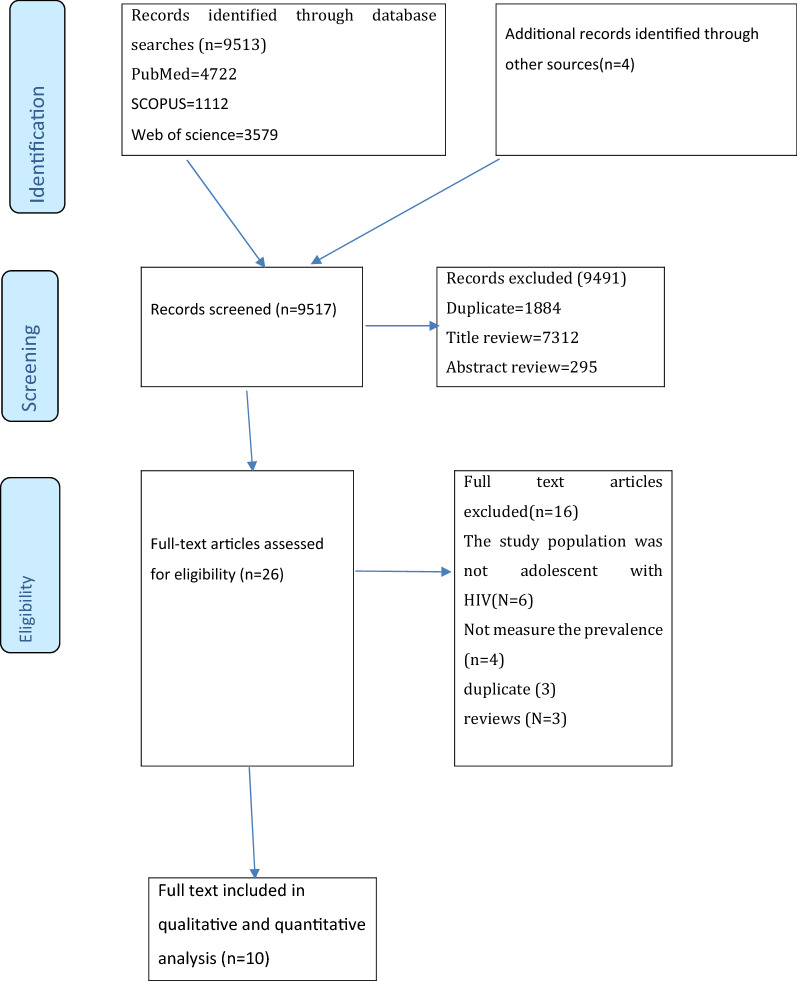
PRISMA flowchart of review search

Our systematic search identified 9613 studies, 1884 of which were duplicates and therefore removed. Further, 7607 records were removed during the evaluation of titles (7312) and abstract (295), as they did not meet the inclusion criteria. Hence, a full-text of 26 publications was retained for further evaluation and 10 of which were qualified for the present systematic review and meta-analysis (Fig. 1).

### Characteristics of included studies

The key characteristics of studies included in this systematic review and meta-analysis are shown in Table 1. A total of 10 articles [3–7, 9, 10, 30–32], with 2642 adolescents living with HIV/AIDS., were included in the final analysis. The vast majority of the included studies were performed in Africa (70%; *n* = 7) and only three studies were conducted outside Africa such as in the USA (n = 1), China (n = 1) and Thailand (n = 1). The studies included in this review were published between 2009 and 2019. The sample size for the included articles ranged from 54 adolescents in Thailand to 566 adolescents in Tanzania.

**Table 1:** The characteristics of studies included in the systematic review and meta-analysis

Study name	Country	Sample size	Data collection tool	Prevalence by age groups	Prevalence, cases (n)	Prevalence by sex
Kemigisha [7]	Uganda	336	CES-D	10–14, 37.4%, 222	45.8%, 154	Male 42.5%, 127
				15–19, 62.3%, 114		Female 47.8%, 207
Musisi et al. [31]	Uganda	85	ICD-10	NA	40.8%, 34	NA
Dow et al. [30]	Tanzania	182	PHQ-9	NA	12.1%, 22	NA
Lee [9]	Thailand	54	CDI	10–14, 23.08%, 9/39	27.8%, 15	Male 22,2%, 6/27
				15–19, 40%, 6/15		Female 33.3%, 9/27
Kim et al. [4]	Malawi	562	CDRS-R	NA	18.9%, 106	Male 15.4%, 38/247
						Female 21.6%, 68/315
Lwidiko et al. [32]	Tanzania	566	CDI	NA	11.5%, 65	NA
Zhou et al. [6]	China	145	CDI	11–14, 23.33%, 7/30	32.41%, 47	Male 33.74%, 28/83
				15–18, 34.78%, 40/115		Female 30.64%, 19/62
Abebe et al. [3]	Ethiopia	353	BDI	15–19, 28.04%, 99/353	28.04%, 99	NA
Lewis et.al. [10]	USA	166	BDI	NA	34.3%, 57	NA
Okawa [5]	Zambia	190	CES-D	15–19, 25.3%, 48/190	25.3%, 48	Male 18.75%, 15/80
						Female 30%, 33/110

*BDI* beck depression inventory; *CDI* children’s depression inventory; *CDRS-R* Children’s Depression Rating Scale; *CES-D* Center for Epidemiologic Studies Depression Scale; *PHQ* patient health questionnaire; *ICD* the international classification of disease

Six instruments were used to assess depression among the participants; the children’s depression inventory (CDI) (3 studies), Center for Epidemiologic Studies Depression Scale (CES-D) (2 studies), beck depression inventory (BDI) (2 studies), patient health questionnaire (PHQ) (1 study), the international classification of disease (ICD) (1 study) and the children’s depression rating scale (CDRS-R) (1 study).

### Quality of included studies

Table 2 shows in the quality and the risk of bias of studies included in this review Four studies have used an adequate sample size (40%) to determine the prevalence of depression. About half of the studies scored positive on the item concerning the response rate (54.54%). To measure depression, all studies utilized a standard instrument or valid diagnostic criteria (97.43%) and all studies (100%) have used suitable statistical analysis to explore the prevalence of depression. Based on the Joanna Briggs institute quality evaluation checklist, the articles involved in the final analysis had a mean quality score of 7.60 ranging from six to nine. Five studies (50%) were high-quality studies (scored 7.60 and above) and the remaining were fair quality articles (scored between 6 and 7.60) (Table 2).

**Table 2:** Qualities of studies included in the systematic review and meta-analysis

Study name	Response									
	Q1	Q2	Q3	Q4	Q5	Q6	Q7	Q8	Q9	Total
Kemigisha [7]	Y	Y	Y	Y	Y	Y	Y	Y	Y	9
Musisi et al. [31]	N	Y	N	Y	Y	Y	Y	Y	U	6
Dow et al. [30]	N	Y	N	Y	Y	N	Y	Y	U	6
Lee [9]	N	Y	N	Y	Y	Y	Y	Y	U	6
Kim et al. [4]	Y	Y	Y	Y	Y	Y	Y	Y	Y	9
Lwidiko et al. [32]	Y	Y	Y	Y	Y	Y	Y	Y	Y	9
Zhuo et al. [6]	Y	Y	N	Y	Y	Y	Y	Y	U	7
Abebe et al. [3]	Y	Y	Y	Y	Y	Y	Y	Y	Y	9
Lewis et.al. [10]	Y	Y	N	Y	Y	Y	Y	Y	U	7
Okawa [5]	Y	Y	N	Y	Y	Y	Y	Y	Y	8
Keys: Q1–Q9 represents questions used to assess the quality of included studies, which are listed below Q1. Was the sample frame appropriate to address the target populations? Q2. Were the study participants sampled appropriately? Q3. Was the sample size adequate? Q4. Were the study subjects and setting described in detail? Q5. Was the data analysis conducted with sufficient coverage of the identified sample? Q6. Was a valid method used in the identification of conditions? Q7. Was the condition measured in a standard, reliable way for all participants? Q8. Was there an appropriate statistical analysis? Q9. Was the response rate adequate, and if not, was the low response rate managed appropriately?										

*Y* yes; *N* no; *U* unclear; *NA* not applicable

### The prevalence of depression among adolescent with HIV/AIDS (meta-analysis)

**Fig. 2:**
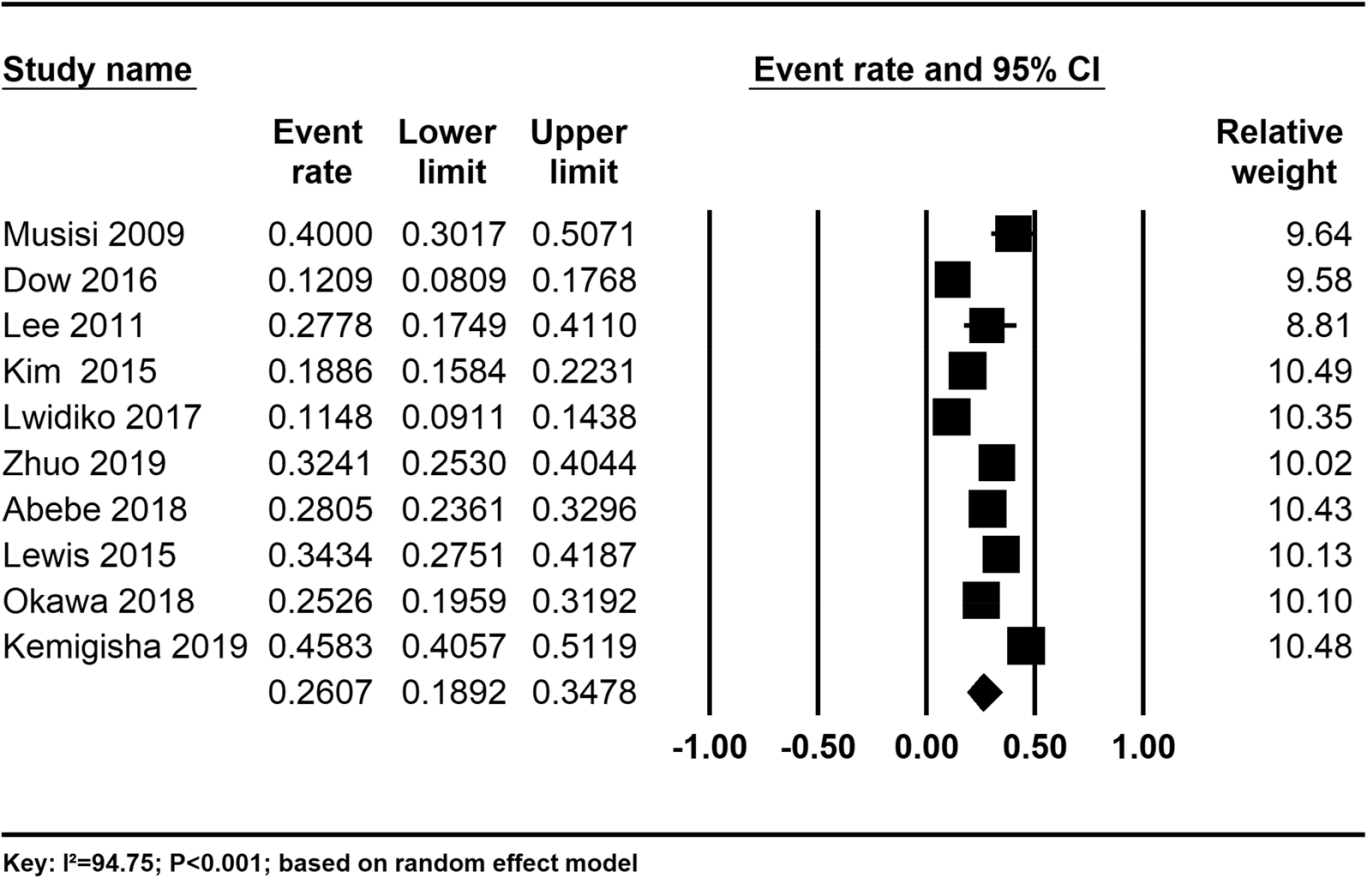
The prevalence of depression among adolescent with HIV/AIDS: a random-effect meta-analysis

The pooled prevalence estimate of depression among adolescents with HIV/AIDS was found to be 26.07% (95% CI 18.92–34.78). There was significant heterogeneity across the studies used for this analysis (*I*^2^ = 94.75%; P < 0.001) (Fig. 2).

The prevalence of depression was 24.27% for high-quality studies and it was 28.14% for fair-quality studies, although the observed difference was not statistically significant (P = 0.630) (Table 3).

**Table 3:** Sensitivity analysis of all studies based on the country of origin, the instrument used, and study quality of the included studies

Subgroups	Studies, n	Prevalence (%)	95% CI	Heterogeneity across the studies		Heterogeneity between groups (*P-*value)
				I^2^	P-value	
Gender						
Female	5	32.15	21.53–45.01	89.68	< 0.001	0.431
Male	5	25.07	14.61–39.56	90.09	< 0.001	
Quality of studies						
High	5	24.27	14.54–37.65	97.07	< 0.001	0.630
Fair	5	28.14		87.06	< 0.001	
Tools used						
BDI	2	30.64	24.94–37.01	52.82	0.145	0.526
CDI	3	22.13	9.91–42.34	94.84	< 0.001	
CES-D	2	35.04	18.00–57.00	95.26	< 0.001	
Others	3	21.64	11.46–37.07	92.58	< 0.001	
Age group						
10–14	3	29.82	20.06–42.83	56.75	0.099	0.414
15–19	5	37.09	24.72–51.43	91.75	< 0.001	

In our subgroup analysis based on the tools used to measure the outcome, we found that the prevalence of depression was high for the studies that used CESD 35.04% (95% CI 18.00–57.00) followed by BDI 30.64% (24.94–37.01) and CDI 22.13% (95% CI 9.91–42.34), although the observed difference was not statistically significant (P = 0.526) (Table 3).

Concerning the sex of the participants, the prevalence of depression was higher for females (32.15%) than males (25.07%) (Table 3).

Moreover, in our subgroup analysis, we found that the prevalence of depression was higher for older adolescents (15–19 years) (37.09%) than younger adolescents (10–14 years) (29.82%) (Table 3).

### Sensitivity analysis

To identify the possible source of heterogeneity across the studies as well as to test the difference across the groups that estimated depression among adolescent, we conducted a stratified analysis by restricting the analysis to the tools used to measure depression (CESD, BDI, CDI and others), the quality of the included studies (high and fair quality), sex (male vs. female) as well as age of the participants (younger and older adolescents). This analysis resulted in the observed variation in the prevalence of depression according to the above four variables (groups) and is not statically significant (P > 0.05) (Table 3).

We also conducted a leave-one-out sensitivity analysis to further examine the possible cause of heterogeneity across the studies involved in the analysis. This analysis suggested that the findings of the main analysis are robust and not dependent on a single study. The pooled estimated prevalence of depression varied between 24.11 (95% CI 17.55–20.33) and 28.39 (95% CI 31.38–36.95) after the deletion of a single study (Additional file 1: Fig. S1).

### Publication bias

**Fig. 3:**
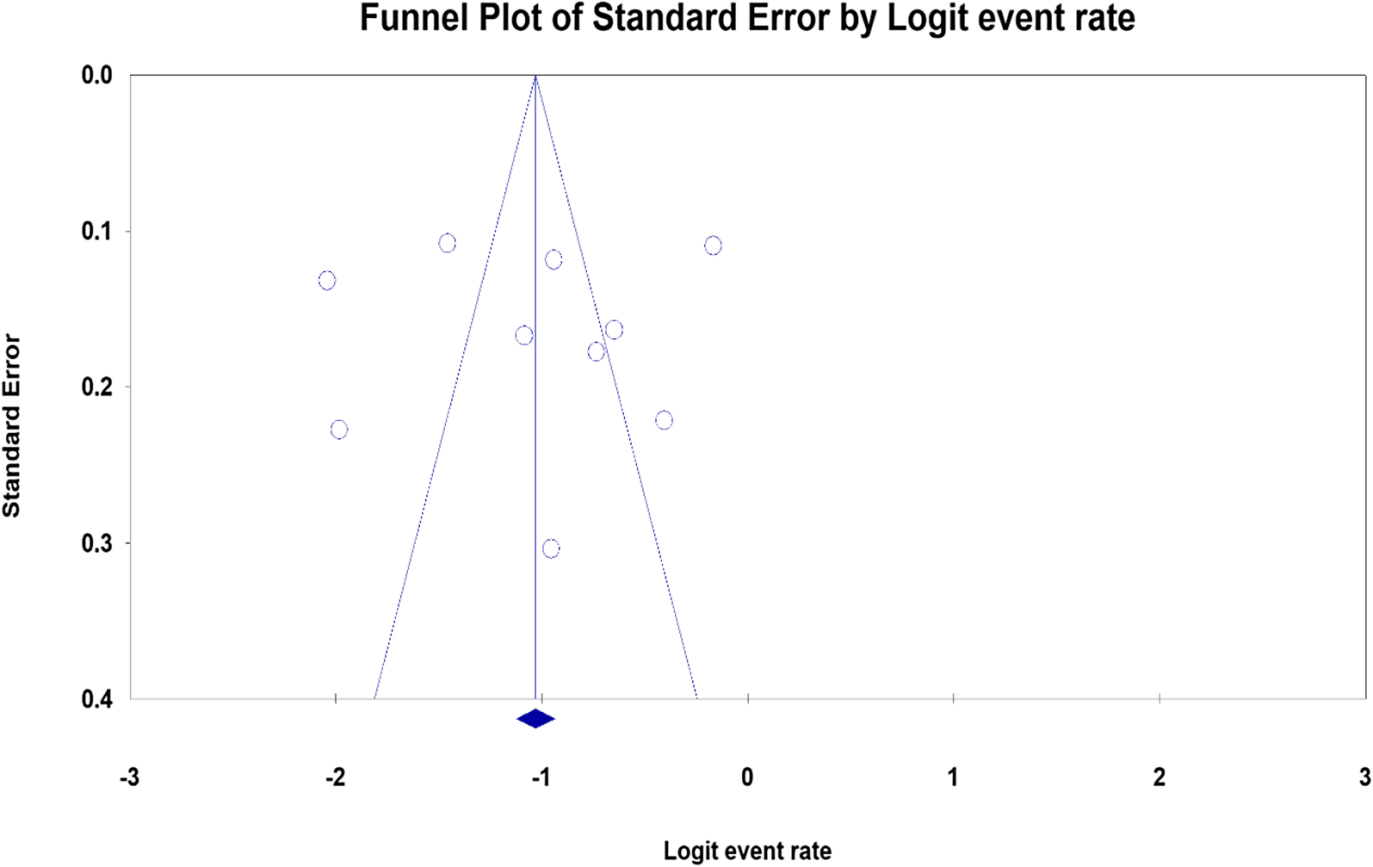
Funnel plot of the risk of publication bias for the prevalence of depression among adolescents with HIV/AIDS

In the current systematic review and meta-analysis, we found no evidence of potential publication bias for the prevalence of depression among adolescents with HIV/AIDS as evidenced by inspection of the funnel plot (symmetric) and results of regression tests associated with the funnel plot (Egger’s test) (B = −552, SE = 4.98, P = 0.914) (Fig. 3).

## Discussion

### Key findings

This study is, to the best of our knowledge, the first systematic review and meta-analysis on the prevalence estimate of depression among adolescents with HIV/AIDS. The review included ten studies that assessed the prevalence of depression among adolescent males and females, which are conducted across seven countries. Our qualitative and quantitative synthesis indicated that the existing scientific evidence on the prevalence of depression among the adolescent demonstrated a considerable variation depending on the countries where the studies are conducted, the gender of the participants, the tools used, the age of the participants, as well as the reported quality of the studies. The vast majority of the included studies were performed in Africa (70%; *n* = 7) and only three studies were conducted outside Africa such as in the USA (n = 1), China (n = 1) and Thailand (n = 1). Roughly half of the included studies reported prevalence by gender (male vs. female) and age categories (10–14 and 15–19 years) and almost all studies used standard instruments to measure the prevalence of depression among the adolescent.

In general, our final meta-analysis demonstrated that a remarkably higher proportion of adolescents with HIV had depression (26.07%). The estimated prevalence was highest amongst female adolescents (32.15%) than males (25.07%) as well as amongst the older adolescents aged 15–19 years (37.09%) than younger adolescents aged 10–14 years (29.82%). These prevalence rates are notably higher compared to that of the general population, suggesting depression is an important and global public health issue among adolescents with depression requiring urgent attention in terms of prevention and treatments.

### Comparisons with the existing evidence

The prevalence estimates of depression among the adolescent in the current study (26.07%), is 2.48 times higher than the reported prevalence of depression in the general population according to a meta-analytic study conducted on the prevalence of depression in adolescents in the general population (10.5%) [33]. This result indicates that depression which might negatively impact the physical, mental and social lives of individuals, is a burning global issue requiring urgent interventions to alleviate the suffering as well as preventions of further negative consequences [34–37]. There are several explanations for the higher prevalence of depression among the adolescent with HIV when compared with the reported prevalence in the general population: one of the possible reason for this variation could be a significant effect of HIV infection on immunity (reduction in CD4 count) which subsequently increase risk of depression in those adolescents with the problem (those who have reduced CDA count), as suggested in previous studies [22, 38]. The higher prevalence rates of opportunistic infections among adolescents with HIV possibly increase the risks of depression are reported in several prior studies [39]. The other potential reason is that adolescents with HIV are at greater risk to experience stigma, discrimination, social association, or marginalization when compared with the general population which is linked with a greater risk of depression among the exposed individuals [40]. The higher prevalence rates of mental disorders that possibly increasing the risk of depression among adolescents could be the other possible reason for the observed differences [41].

As expected, our study showed that the prevalence of depression was highest in females than males and the older adolescents than younger adolescents, which were in agreement with the reported prevalence rates of depression from adolescents in the general populations, cancer patients, as well as refugees and asylum seekers and [42–44]. The possible reasons for the gender differences in the prevalence of depression include adverse experiences in childhood and adolescence, the sociocultural roles of adverse events (psychological trauma), sexual abuse and the variations in coping skills [45, 46]. The biological and genetic variations could be also mong the possible contributing factors for the observed variation [47].

The current systematic review and meta-analysis have found considerable heterogeneity across the studies that determined depression among adolescents with HIV. The observed heterogeneity could be due to the variation in the characteristics of the participants as well as the methodology of the included studies. Regarding the methodological differences, the include studies varied according to the sample size, the tools used to estimate the outcomes, the sampling produces, as well as the source of population. Additionally, the included studies varied according to the gender of the participants, the location where the participants have resided and they are selected from seven countries with certain variations in socioeconomic and cultural backgrounds affecting the mental health of the participants. To identify the possible source of heterogeneity across the studies we conducted stratified analysis by restricting the analysis to the tools used to measure depression (CESD, BDI, CDI and others), the quality of the included studies (high and fair quality), sex (male vs. female) as well as age of the participants (younger and older adolescents) and we observed no significant variation in the prevalence of depression across the groups (P > 0.05).

### Strength and limitations

The present study has several strengths. First, being the first systematic review and meta-analysis to determine the prevalence of depression among adolescents with HIV/AIDS. Second, estimating the prevalence rates of depression with regards to the specific gender and age categories. Third, performing a subgroup and sensitivity depending on the origin of the study, the instruments used to estimate depression, as well as the quality of the studies to detect the possible risk of bias.

Several limitations of this systematic review and meta-analysis should be considered. First, the vast majority of the included studies were conducted in developing countries particularly in Africa so that the reported prevalence of depression in the current study may not represent the existing prevalence in the developed countries; second, we have included studies published English language, which suggests that potential studies conducted in other languages might be missed.

### The implication of the findings

The current review has many research and clinical implications. First, future studies are needed to investigate the possible reasons for the higher prevalence rates of depression among adolescents with HIV as compared to the reported estimates in the general population. Second, further studies from high and middle-income countries are imperative. Third, early screening and intervention of depression in adolescents with HIV based on coordinated and integrated public health approaches are required to alleviate the suffering and reduce further negative consequences. Fourth, in this meta-analysis virtually all the included studies have used screening instruments to measure depression or reported depressive symptoms rather than diagnosed disorders (requires diagnostic instrument). Thus, future studies into the prevalence of depressive disorders are needed.

## Conclusion

This review demonstrated that the prevalence of depression is significantly higher among adolescents with HIV/AIDS, suggesting the benefits of early screening and intervention of depression among those population groups. Further studies investigating the potential reasons for the higher prevalence estimates of depression among adolescents with HIV/AIDS are needed. In addition, studies investigating better mechanisms of screening, prevention and interventions of those problems among adolescents with HIV are needed.

## Supplementary Information

**Additional file 1: Figure S1.** Snapshot of the details of serch terms used in the three databases—PubMed, Scopus and Web of Science.

## Data Availability

All data generated or analyzed during this study are included in this article.

## Abbreviations

BDI: Beck depression inventory
CDI: Children’s depression inventory
CDRS-R: Children’s Depression Rating Scale
CES-D: Center for Epidemiologic Studies Depression Scale
PHQ: Patient health questionnaire
ICD: The international classification of disease
NOS: Newcastle–Ottawa scale
PRISMA: Preferred reporting items for systematic reviews and meta-analyses
USA: United States of America
