# Supplementary figures and images for: The prevalence of depression among adolescent with HIV/AIDS: a systematic review and meta-analysis
Source: AIDS Res Ther. 2021 Apr 27;18:23. doi: 10.1186/s12981-021-00351-1 (PMC8077927; doi:10.1186/s12981-021-00351-1)

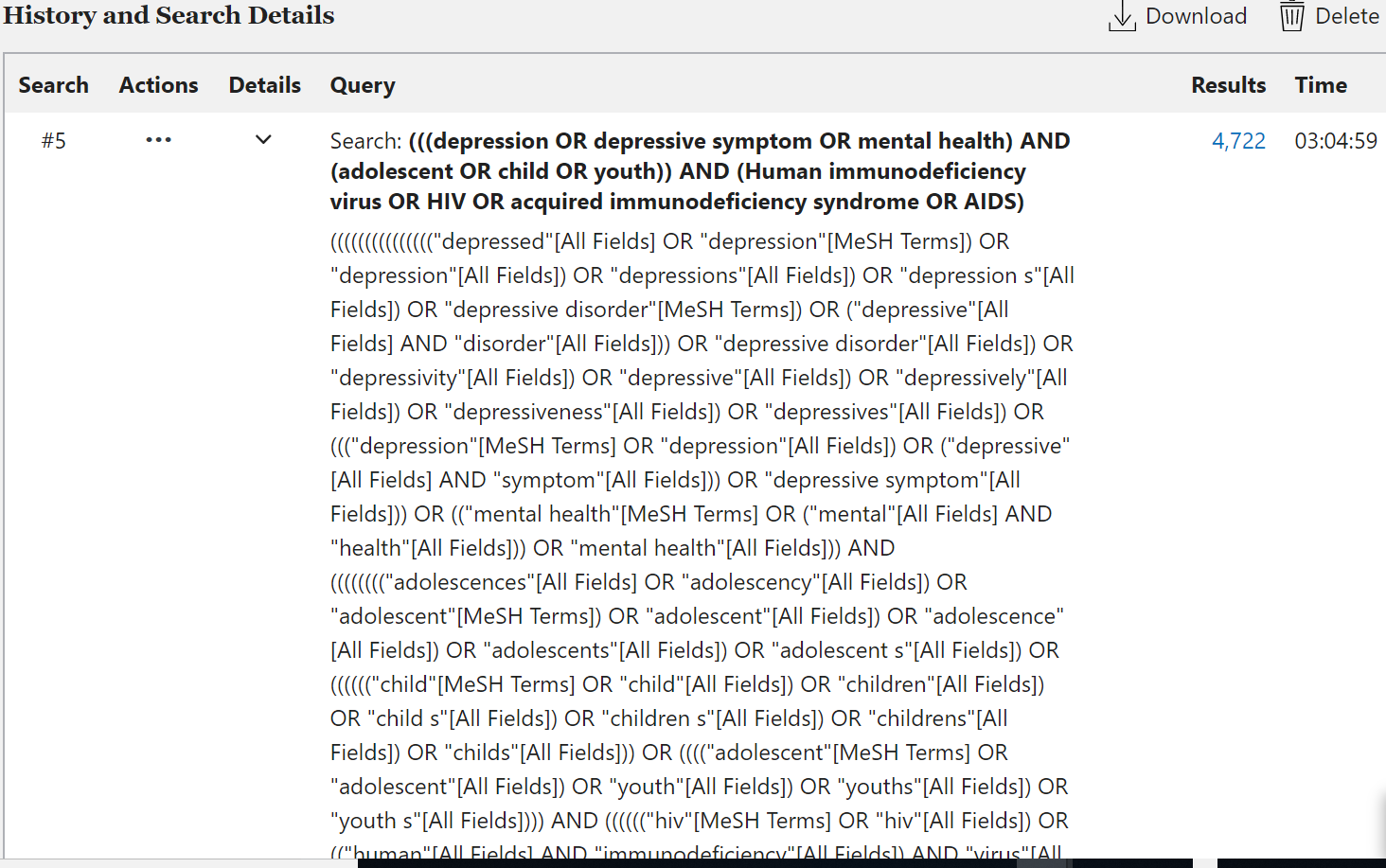

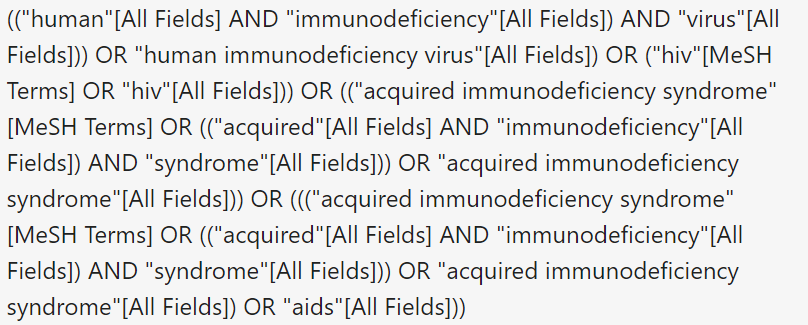


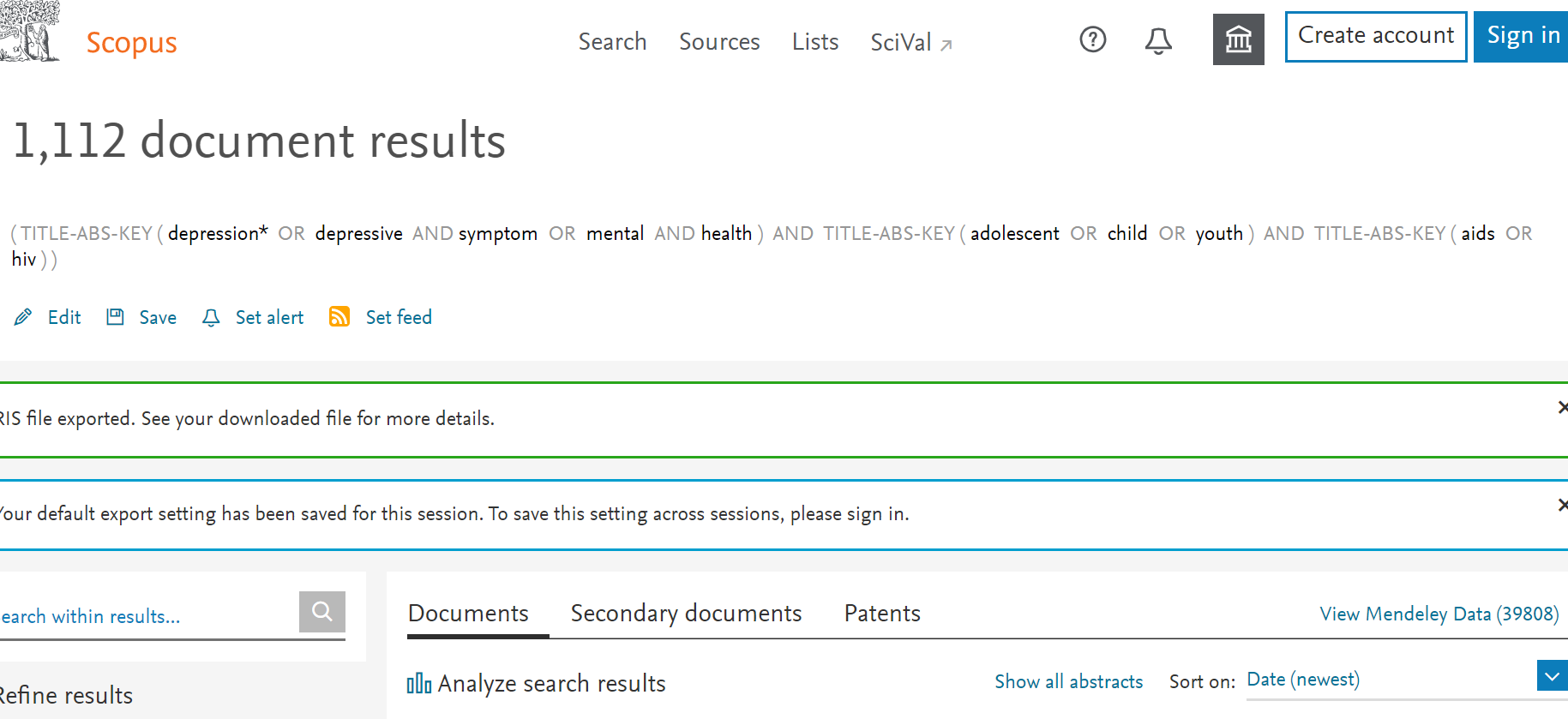


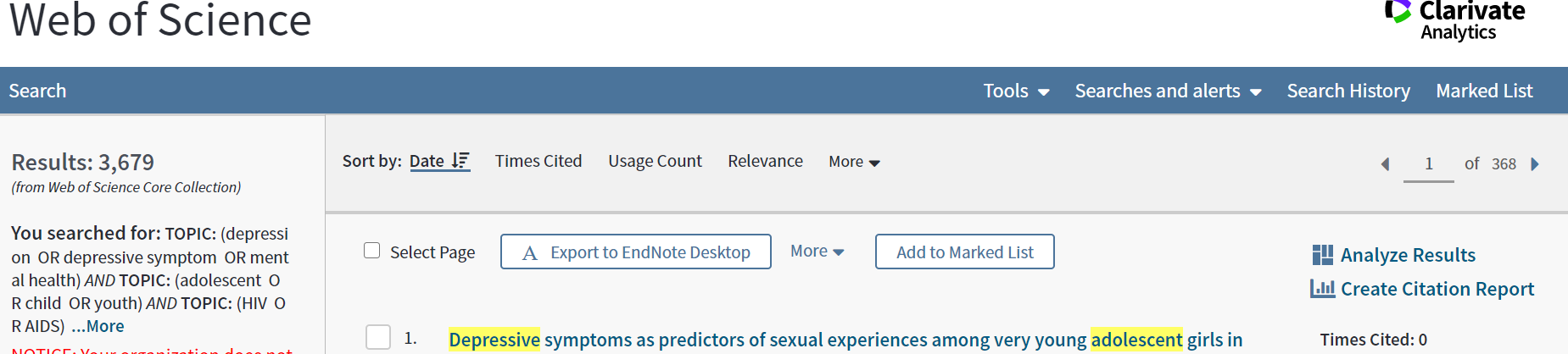

Supplement: Supplementary file 1 — Additional file 1: Figure S1. Snapshot of the details of serch terms used in the three databases—PubMed, Scopus and Web of Science. [file 12981_2021_351_MOESM1_ESM.docx]
